# Toward universal human papillomavirus vaccination for adolescent girls in Hong Kong: a policy analysis

**DOI:** 10.1057/s41271-020-00220-7

**Published:** 2020-02-13

**Authors:** Ruirui Chen, Eliza Wong, Lijuan Wu, Yuanfang Zhu

**Affiliations:** 1 Jinan University-affiliated Shenzhen Baoan Women’s and Children’s Hospital, Shenzhen, China; 2 Division of Health System, Policy & Management, School of Public Health and Primary Care, The Chinese University of Hong Kong, Shatin, Hong Kong

**Keywords:** HPV vaccination, Universal coverage, Health policy analysis, Hong Kong, Stakeholder analysis

## Abstract

Studies have assessed early population-level impact of human papillomavirus (HPV) vaccination programs for preventing cervical cancer. Through a case study in Hong Kong we examined stakeholder engagement and interactions to promote a universal HPV vaccination program using the Health Policy Triangle framework for structured health policy analysis. Using data from a document review and semi-structured in-depth interviews, we used thematic and stakeholder analyses to describe the process of policy formation. Given Hong Kong’s political and health system, and a mix of Chinese and Western values, stakeholders judged legitimacy of the process differently. We discuss their varied ethical stances and the role of research evidence for informing policy-making. For effective HPV vaccination policy and promotion of universal free HPV vaccination among adolescent girls, new strategies are needed to broaden acceptance of the process, to frame policies in terms of facts and values, and to connect research to policy-making and improve coalition-building.

## Introduction

Countries with sufficient resources have successfully implemented human papillomavirus (HPV) vaccination programs with high rates of uptake among adolescent girls [1]. A recent systematic review assessed early population-level impact on reductions in HPV vaccine-type prevalence, genital wart diagnoses, pre-cancer high grade cervical lesions, and herd immunity [2]. A World Health Organization (WHO) position paper in 2014 recommended that all countries should try to integrate HPV vaccine into their national immunization programs to prevent cervical cancer or other HPV-related diseases, and to ensure that vaccination is programmatically feasible, cost-effective, and sustainable [3]. In Australia, HPV vaccination is a part of routine school-based immunization program, delivered free of charge for all sexes aged 12–13 years. In the United States, case studies delved into the politics of HPV vaccination policy formation [4, 5]. Other studies examined HPV vaccination in Asian countries, including issues such as financing, policy development, and the feasibility of a government-implemented program [6–8]. Still lacking, however, was a structured analysis of political dimensions of policy-making, in which the use of frameworks could guide analysis, deepen understanding, and support generalization to other settings targeting universal HPV vaccination. Walt and Gilson developed the framework Health Policy Triangle to examine how context, actors, content, and processes interact to shape policy-making. Researchers have used this framework to analyze other health issues, including health sector reform, reproductive health, and vaccine introduction [9].

Disease burden of cervical cancer remains a concern for women’s health in Hong Kong. The age-standardized incidence rate of cervical cancer was 8.4 per 100,000 females in 2015 when 500 incident cases and 151 deaths occurred, meaning it ranked the seventh among common female cancers and ninth for major causes of cancer deaths, respectively [10]. Although Hong Kong introduced an organized cervical screening program in 2004, no apparent decline in age-standardized incidence appeared. And these features persist since the program began:

- about 65% of women aged 25 to 64 had ever undergone screening for cervical cancer,
- low registration rates of physicians to administer it,
- among eligible women, inappropriate over-screening of low-risk women,
- a lack of targeted interventions for those at risk [11–13].

Therefore, clinical implications of HPV vaccines may be notably beneficial in Hong Kong where the cervical screening program is not functioning as planned. Hong Kong bases immunization policy-making on a standard two-round evaluation by the Centre for Health Protection. The first round of evaluation is made by the Scientific Committee on Vaccine Preventable Diseases, established in 2004 to provide science-based advice on vaccine use at the population level. The second round is an administrative feasibility evaluation including analysis of costs and benefits, human resources capability, and public acceptability [14]. Hong Kong approved the use of HPV vaccine in 2008, but has not yet initiated a universal free-of-charge HPV vaccination program for adolescent girls due to social instability starting from May 2019, although the Chief Executive of Hong Kong Carrie Lam proposed it in a policy address in October 2018 [15].

We aimed to identify activities undertaken by stakeholders to advocate for a new policy of universal HPV vaccination for adolescent girls in Hong Kong, particularly on political dimensions of policy-making. A universal program indicates the integration of HPV vaccine into the national immunization program to offer free-of-charge vaccination to girls aged 12 years old for cervical cancer prevention. We focused on two questions: (1) What organizations and categories of people engaged in policy formation? (2) What factors influenced the policy-making process? For this study, we adapted the Health Policy Triangle framework to systematically review policy-making in terms of context, actors, process, and content [9].

## Methods

Our research team collected information in two ways: (1) a formal review of related documents, press releases, and technical reports on public websites; and (2) qualitative in-depth interviews with the key informants who could influence the direction and outcome of the policy [16–18]. We searched the official websites of Hong Kong government and ministries of health and other involved organizations, using broad terms “HPV vaccination” or “HPV vaccine.” Based on the collected and reviewed documents, we developed two interview guides to capture insights into actors’ perceptions and motivations of those whom we identified to have been involved as actors in or close observers of the policy process. We call these people (and their organizations) ‘stakeholders.’

We used purposive sampling to explore opinions and perceptions of these stakeholders. We have published details and methodological limitations of our sampling approach elsewhere [19]. We obtained ethical approval from the Survey and Behavioral Research Ethics Committee of the Chinese University of Hong Kong. We conducted face-to-face interviews with 16 individuals after obtaining their consent and collected five written responses, all in the period April to September 2016. A pharmaceutical company provided one of the written responses; health departments completed the other four. The interviews ranged in duration from 26 to 56 min. We audio recorded and transcribed the interviews verbatim.

We used stakeholder analysis and thematic analysis in accordance with the Health Policy Triangle framework. For the stakeholder analysis, we developed a matrix to display the stakeholders’ positions, power, and interests. For each stakeholder we display the then current level of knowledge and power and noted whether or not each stakeholder supported a universal program for 12-year-old girls. We rank each respondent on a five-point scale (1 as the minimum and 5 as the maximum) [20, 21]. To increase the validity of the scale, we adjusted self-reported power using the interpretation of other stakeholders as to the level of influence of the others. We further adjusted these judgments based on secondary data from documents we reviewed.

For thematic analysis, we coded line-by-line, combined statements describing the same phenomenon into themes, and then compared the themes generated with pre-defined concepts and categories in the interview guide to allow emergence of new themes.

## Results

**Fig. 1:**
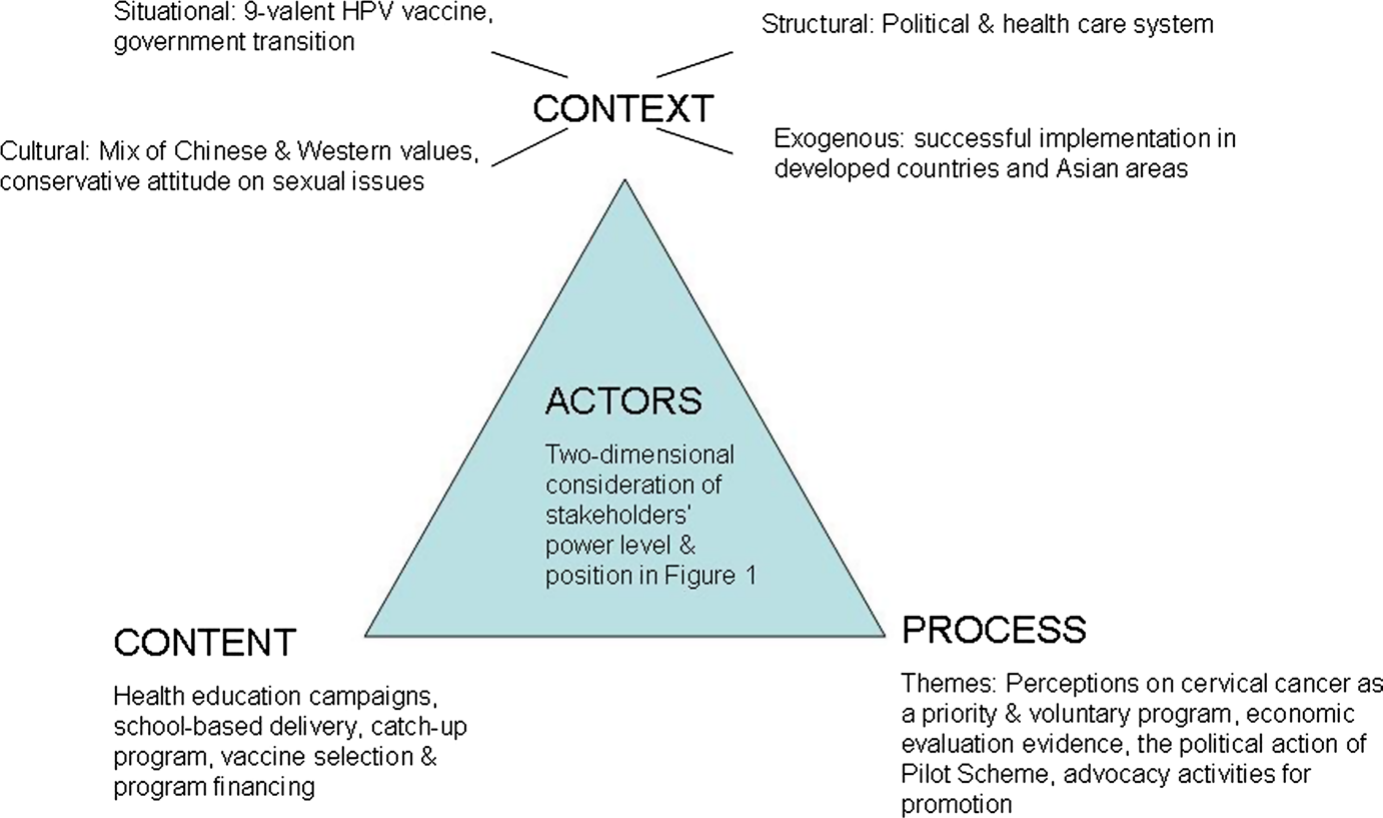
Health policy triangle framework: summary of findings

Figure 1 depicts our main findings using the framework. We present findings in the following four sections of text: context, actors, policy process themes, and policy content.

### Context

To facilitate understanding of the policy environment, we categorized contextual factors as ‘situational,’ ‘structural,’ ‘cultural,’ and ‘international’ because any nation's public policies can be explained by these factors [22]. After the transfer of sovereignty over Hong Kong from Britain to China in 1997, the city became an autonomous Special Administrative Region of the People's Republic of China, with one exception: defense and foreign affairs remained under a framework known as “one country, two systems” and the Basic Law is its constitutional source of authority [23].

*Situational factors* are those transient or idiosyncratic conditions sometimes called ‘focusing events.’ The Pharmacy and Poison Board of Hong Kong approved the new nonavalent HPV vaccine to cover more virus types in early 2016. The other focusing event was the once-only occurrence of government transition in Year 2016 and 2017—from the Chief Executive Leung Chun-ying to Carrie Lam. This may have influenced the way authorities put policy proposals on the agenda.

A Chief Executive and an Executive Council lead Hong Kong’s government. The Legislative Council is the law-making body, and in it the Democratic Alliance for the Betterment and Progress of Hong Kong is the largest party. The Food and Health Bureau formulates policies and carries out decisions in health care system. The Department of Health is the Government's health adviser and executing agency for health policies and statutory functions. A well-functioning health care system, including school-based vaccination infrastructure and a drug evaluation mechanism, demonstrates Hong Kong’s government’s capacity; it enjoys confidence of the public. Hong Kong benefited from its political stability in 20 years from 1997 to 2018 and is a society with high level of free-market economic development and great participation of civil society organizations.

*Cultural factors* include a mix of Chinese and Western values emanating from long-term influence of the West and public acceptance of immunizations as preventive measures. The attitude about sexual issues is conservative because Chinese culture in this population includes deeply rooted Confucianism [24].

*International influence* means a majority of stakeholders have drawn on successful experiences of developed countries such as the United Kingdom and Australia that enjoy similar economic prosperity. Although few areas or countries in Asia have universal coverage, Hong Kong’s geographical characteristics explain the special attention to Macau’s universal free-of-charge program for system similarity and geographical closeness. Hong Kong also noted Japan’s withdrawal of a national immunization program due to side effects reported in mass media. In the media reports, the head of Nationwide Cervical Cancer Victim Liaison Committee claimed compensation from the government for her daughter’s suspected complex regional pain syndrome and loss of walking ability caused by HPV vaccine injection; this gained considerable public attention in Japan [25].

### Actors

**Fig. 2:**
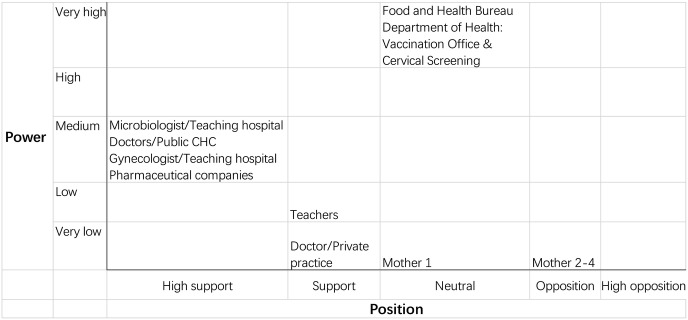
Hong Kong stakeholders in force-field mapping. Stakeholders at same level of position and power appear as a group. *CHC* community health center, *NGO* non-governmental organization, *FPA* family planning association

We present stakeholders’ roles, interests, knowledge levels, self-reports of power (adjusted), and their positions in Table 1. We map the estimated power of each stakeholder based on the position of each—to indicate their potential influence (Fig. 2, Force-field mapping) [26]. We did not identify any opponents such as religious or anti-vaccine groups in document review or interviews of stakeholders. The highest level of influence over policy formation came from administrative authorities in health care system despite their ‘policy neutrality’; that is, they neither supported nor opposed the proposed universal program. Most stakeholders operated from within government institutions except for private family doctors. Although some of the doctors had connections with health departments because they provided technical assistance as consultants, their involvement was more limited as they operated only as individuals without institutional leverage. Private family doctors, teachers, and mothers had low levels of power level because they were not organized to exert influence collectively. Despite their limited impact on policy formation, they were a dominant source of engagement in implementation. We identified no coalition during the interviews.

**Table 1:** Analysis of Hong Kong stakeholders’ role, interest, knowledge level, power, and position

Interviewee	Stakeholder role	Stakeholder affiliation	Stakeholder interest	Knowledge	Power/adjusted power	Position
1	Doctor	Public community health center (CHC) and academic institution	Health promotion in schools	5	1/3	5
2	Doctor	Teaching hospital	Microbiology	5	2/3	5
3	Doctor	Public CHC nd academic institution	Public health	5	2/3	5
4	Doctor	NGO: Family Planning Association (FPA)	Gynecology	5	2/2	5
5	Doctor	Private practice	General practitioner	4	1/1	4
6	Doctor	NGO: Non-profit CHC	General practitioner	5	2/2	5^a^
7	NGO representative	CEO of Karen Leung Foundation	Cervical cancer prevention	4	2/3	5
8	Doctor	Teaching hospital	Gynecology	5	2^b^/2	5
9	Supplier representative	Pharmaceutical company: Merck & Co	Profit and raising awareness	4	1/2	5
10	Supplier representative	Pharmaceutical company: Glaxo SmithKline Biologicals	Profit and raising awareness	4	0^c^/2	5
11	Teacher 1	Middle school	Raising awareness^d^	3	2/2	4
12	Teacher 2	Primary school	Raising awareness^d^	1	2/2	4
13	Mother 1	Home	Decision maker for vaccine recipient	3	1/1	3
14	Mother 2	Home	Decision maker for vaccine recipient	2	1/1	2
15	Mother 3	Home	Decision maker for vaccine recipient	1	1/1	2
16	Mother 4	Home	Decision maker for vaccine recipient	3	1/1	2

Stakeholders’ knowledge, self-reported, and adjusted power was attributed on a five-point scale (1 = very low; 2 = low; 3 = medium; 4 = high; 5 = very high); stakeholders’ position was attributed on a five-point scale (5 = high support; 4 = support; 3 = neural; 2 = opposition; 1 = high opposition)

^a^High support for targeting 15-year-old girls

^b^Self-reported low power level of 1 or 2

^c^No response on power level in a written reply

^d^Self-reported interest

The two pharmaceutical companies reported to have played a limited role even though they possessed financial and other resources. Medical professionals had to divulge potential conflicts of interest to their colleagues and the public if their relationships and behavior might increase vaccine sales. Stakeholders reported that doctors’ influence could have been weakened if they were known to advise the pharmaceutical companies selling the HPV vaccine. Stakeholders told us that non-profit health organizations and academics held cautious views about cooperation with the industry on research related to HPV vaccination. Several stakeholders denied the industry’s invitation to conduct research together, saying they had avoided potential negative impact on the credibility and objectivity of the research to maximize public benefit. Accordingly, the pharmaceutical representatives reported their limited scope for industry involvement through these interactions with non-profits and academics.

### Themes in policy process

#### Theme 1: Perceptions about cervical cancer as a priority

Most stakeholders perceived cervical cancer prevention to be a high priority despite it not placing as a top health concern among women. The respondents used objective measures for justification of priority, including rank in cause of death, incidence rate, and number of cases diagnosed. A gynecologist reported that static incidence made cervical cancer no longer ‘eye-catching’ due to increasing incidence of other cancers in Hong Kong’s aging population. Some of the stakeholders also used comparisons with other developed countries’ incidence rates to determine priority of cervical cancer. These credible indicators provided a basis for the participant stakeholders to provide the assessments of their own position and level of power. The most often highlighted feature of cervical cancer was its susceptibility to prevention using the existing government-subsidized screening program. Interviewees without medical background tended to relate cervical cancer prevention to fertility and suggested that eligible women would prioritize cervical cancer prevention through regular screening.

#### Theme 2: Perceptions about voluntary HPV vaccination

Participants expressed mixed perceptions about voluntary HPV vaccination. An example of a positive opinion reported is “an active introduction with increasing awareness”; a negative one is “a complete failure” because the uptake rate in 10 years’ time of availability remained low. Most commented that family doctors did not contribute much to the success of this voluntary program by connecting patients with community health centers due to their low level of public health awareness about cervical cancer prevention. Interviewed mothers stated that mostly government action influenced their behaviors. Participants in the study cited absence of a visible strategy to promote HPV vaccination to have created a lack of confidence in the program, and confusion about what was expected of them.

#### Theme 3: Economic evaluation evidence

Several stakeholders emphasized the importance of economic evaluation as did all those who responded in writing from the health departments. Modeling estimates of the incremental cost-effectiveness ratio appear to be substantially below the criteria of per capita gross domestic product, thus justifying the vaccination of 12-year-old girls as a high value-for-money intervention [27]. However, stakeholders who valued the economic evaluation evidence varied in their assumptions, interpretations, and conclusions because they also differ in how they understood cost-effectiveness, based on comparing money invested and money saved from averted cancer cases. The stakeholders considered vaccine price to be the most controversial part; cost was the major driver for determining the ratio, but it was not fixed in a tiered pricing system when developed countries achieved low tender prices. The research team at the University of Hong Kong that completed the cost-effectiveness analysis in 2012 later conducted a cost benefit analysis and disseminated it at an academic conference of the Hong Kong Academy of Medicine in June 2017 [28].

#### Theme 4: The political action of pilot scheme

The Chief Executive Leung Chun-ying proposed a pilot scheme of providing free cervical cancer vaccination to eligible low-income girls in the Policy Address of 2016 before we started the interviews. The Community Care Fund under the Commission on Poverty launched this scheme and commissioned the Hong Kong Family Planning Association to provide free vaccination services to girls aged 9 to 18 who received Comprehensive Social Security Assistance, and to female students aged 9 or above who received full grants under the School Textbook Assistance Scheme [29]. The Scientific Committee on Vaccine Preventable Diseases posted a consensus statement on the use of HPV vaccine to prevent of cervical cancer on the government website in September of 2016, just before the start of the pilot scheme [30]. The CEO of Hong Kong Family Planning Association saw mounting of a program as a first organized course of action before a probable wide-scale program. Several stakeholders expressed concern from an epidemiological perspective about limited effectiveness of the pilot scheme at population level if no herd immunity was achieved. Also they found little evidence that the targeted girls were at high risk for HPV infection. Thus, they raised concerns about potential stigmatization of girls—based on an assumption that they were likely to be sexually active, or “labeling these underprivileged girls” given the conservative attitude about sexual activity among local population.

Interviewee 1 (Table 1) saw the pilot scheme as a politically motivated policy “on the radar screen” and commented that initiation of such a pilot scheme was reasonable from the public health viewpoint and that use of available funding from the Community Care Fund could make approval by the Legislative Council easy and quick. Another stakeholder commented that it seemed odd to make incremental changes in an immunization program, piloting in particular population or in particular places because she thought immunization program should cover adolescent girls in general.

#### Theme 5: Advocacy efforts to promote universal coverage for target girls

Several efforts to promote HPV vaccination have taken place. The vaccine industry, led by a private gynecologist, launched a first alliance early, when the private sector played an important role in the first several years between 2008 and 2012. Then two NGOs organized immunization service provision in local schools. One of the NGOs reported their efforts to communicate results with government health departments upon completion of their final report. Members of a committee of the Hong Kong College of Obstetrics & Gynecology also wrote a letter to the health departments, the only action taken by professional bodies. The government replied to this group, but these stakeholders had seen no government action by the date of our interview in 2016.

The largest party in Legislative Council paid some attention to HPV vaccination. Its Women’s Affairs Committee, in late 2013, expressed concern about provision of free HPV vaccines first to “under-privileged” girls before extending free vaccine to all adolescent girls. Early in 2014, the Women’s Affairs Committee organized a press briefing to catch attention of the Chief Executive of Hong Kong before the release of that year’s policy address [31, 32]. Mass media played a pivotal role in shaping issues for public debate. Andrew Work argued in a commentary that the “political risk” for the Hong Kong government for not having a cost-effective school-based program would be to show that the government had not cared to protect women, and thus it was not aligning Hong Kong with modern nations for HPV vaccination [33]. A local newspaper, the South China Morning Post, later published two reports around the time of the launch of the pilot scheme urging the Hong Kong government to provide a public program for all girls [34, 35]. A more recent report presented on state-run Radio Television HK in April 2017 recorded establishment of HPV Prevention Alliance by medical professionals to raise awareness of the public [36].

### Policy content

Respondents viewed health education campaigns to be the most critical action for increasing awareness among the general population to bolster initiation of a universal program. Respondents also reported as important the extension of the ‘Pilot Scheme’ to cover more of the targeted girls along with professional training sessions to disseminate the latest evidence.

Despite of concerns about increasing workload and coordination with the education department and schools, stakeholders preferred school-based service delivery to achieve high parent acceptance and uptake rates. Respondents reported that Department of Health needed more evidence for co-administration of HPV with a booster dose of diphtheria and tetanus toxoids, acellular pertussis, and inactivated poliovirus vaccine for 12-year-old girls in schools. We found a lack of consensus on the best age at which to immunize, but respondents said that health education tailored for adolescents was essential to gain their assent.

## Discussion

HPV vaccination policy-making emerged from the internal and political dynamics of Hong Kong and its broad political and social environment. Whether to provide a universal HPV vaccination program free-of-charge drew high political interest over time and engagement of stakeholders beyond health system [37].

First, the legitimacy of the policy-making process for HPV vaccination in the eyes of our research team depended, in part, on who made the recommendation and when to initiate discussions on the policy change. Views synthesized in the consensus statement issued by the Scientific Committee on Vaccine Preventable Diseases could be explained by ‘Solutions seeking problems.’ By this we mean a situation in which solutions offered to the problem on HPV vaccination are not related to solving the problem—cervical cancer prevention [38]. Preferences for particular policies could be fulfilled to influence policy-making by selecting particular evidence and participants for solutions looking for issues to which they might be the answer. Hence, gathering talents from more types of medical professionals than those involved in infectious disease prevention, including experts in cancer prevention and gynecology, may prove more convincing to medical professionals and the public in the next round of discussion. Alternatively, regarding the timing of issuance of the latest consensus statement, questions may be raised about whether it was more likely to be event-triggered, not planned. Neither a WHO recommendation nor a newly issued vaccine product served as a prompt to initiate discussion; instead the Scientific Committee on Vaccine Preventable Diseases prepared and issued a consensus statement to generate a scientific recommendation to facilitate implementation of a pro-poor HPV immunization policy.

Second, varied positions of the stakeholders on universal HPV vaccination represent their ethical stances. We categorized them in four groups: subject utilitarians, objective utilitarians, egalitarian liberals, and communitarians [39, 40]. Subject utilitarians dominated the process. They focused on consequences, judging policy by the sum total of individuals whose well-being in a society would improve. The latest consensus statement emphasized such population benefits. Objective utilitarians could use the projected quality-adjusted life year evaluation in the earlier cost-effectiveness analysis as a measure. Egalitarian liberals proposed the pilot scheme. They saw immunization as an expensive sort of health service. Other egalitarian liberals held the view that government should ensure a minimum quantity and quality of health services, and that HPV vaccination should be among them. We identified a communitarian view on sexually transmitted infection prevention. That means asking the potential target population to adhere to a community norm for healthy behavior (safe sex) to produce a good society, along with the protection afforded by HPV vaccines. Chinese culture influenced by Confucianism embraces this view [41]. The combination of Western and Eastern culture in Hong Kong could explain why the consensus statement drew from two ethical perspectives: subjective utilitarianism and communitarianism.

Third, reframing HPV vaccination to argue facts and values helped avoid confusion and misunderstanding about the policy change. In the view of the public, HPV vaccination was related to sexually transmitted disease prevention. Therefore, presentation of facts on HPV vaccination should revolve around its role as one of primary-level interventions for cervical cancer prevention, a remaining priority in women’s health based on a comprehensive review of comparative indicators, trends in incidence, as well as potential impact on screening and treatment. Along with admitting safe sexual behaviors are necessary to prevent sexually transmitted diseases and cervical cancer, it will be useful to point out that HPV vaccination is also a positive way to protect oneself. This is consistent with suggestions from the study on the politics in six states of the US to avoid battles motivated by morality and “cultural war” issues [4].

Fourth, our study suggests that unlike the situation in low- and middle-income settings, there was acknowledgement of the important role of research evidence in HPV vaccination policy-making here [19]. Hong Kong’s researchers had the capacity to do studies on disease burden and economic evaluations, and local decision makers recognized the importance of evidence. Researchers (from a prestigious institution) presented a high quality of cost-effectiveness analysis, although no support for their views had been published in journals. Low level of certainty about whether a universal program is cost-effective and a lack of communication between researchers and policy-makers, however, left the evaluation open to interpretation. Some government officials perceived a universal program would not be cost-effective. The same difficulty may compromise effective use of cost–benefit analysis results. Therefore, more efforts to improve dissemination and communication of evaluation results to more types of professionals, and to related government departments, will be needed to avoid misunderstanding and convince the influential subjective utilitarians. This is consistent with previous studies on evidence-informed vaccine introduction targeting dissemination of research findings to laypersons and government departments [42–44].

Finally, characteristics of the previous alliance between NGOs and professionals, including that the parties were only loosely connected and cooperated only intermittently on a small-scale, partially explain the failure in policy advocacy to achieve a universal program. Local NGOs, whether charity funding organizations or service providers, conducted substantial work to improve public awareness through education. Their efforts, however, were insufficient to influence policy-making. Their scale was small and prior cooperation with government involved only service provision, not policy formulation. If the medical professionals in different institutions, whether public, private, or non-profit, could organize themselves in professional bodies, their power to influence policy may increase. However, this transition will not elevate them to primary drivers of this policy. Although specialists formed the HPV Prevention Alliance in April 2017 to raise public awareness, a larger coalition to engage a wider range of stakeholders will be needed to gain sufficient power. Only organized forces and greater unity of these supporters could effectively influence the policy agenda [45]. A window of opportunity for policy change opened when evaluations of the pilot scheme appeared in April 2018 and a new government seeking proposals was about to make key decisions.

Our study has limitations. The respondents interviewed may not be a representative sample of all stakeholders that could influence the policy process. Also, health authorities, very important actors, only provided short written response and did not answer the questions on position and power level in the interview guide. These respondents’ perceptions may be different from the opinions of their organization. Future study could focus on stakeholder engagement and interactions during policy implementation with a repetitive stakeholder analysis to investigate the change of actors’ positions and levels of power.

## Conclusion

Multiple stakeholders’ involvement and their underlying values and ethical perspectives indicate the complexities of policy-making to achieve universal, free-of-charge HPV vaccination in Hong Kong. The effectiveness of advocacy to foster development of effective HPV vaccination policy depends upon strategies to improve the legitimacy of policy-making, how well HPV vaccination is framed, better alignment of research to the needs of policy-makers, and coalition-building.
